# New Targeted Therapies and Immunotherapies for Locally Advanced Periocular Malignant Tumours: Towards a New ‘Eye-Sparing’ Paradigm?

**DOI:** 10.3390/cancers13112822

**Published:** 2021-06-05

**Authors:** Arnaud Martel, Sandra Lassalle, Alexandra Picard-Gauci, Lauris Gastaud, Henri Montaudie, Corine Bertolotto, Sacha Nahon-Esteve, Gilles Poissonnet, Paul Hofman, Stephanie Baillif

**Affiliations:** 1 Department of Ophthalmology, University Hospital of Nice, Cote d’Azur University, 06000 Nice, France; nahon-esteve.s@chu-nice.fr (S.N.-E.); baillif.s@chu-nice.fr (S.B.); 2 FHU OncoAge, Institute for Research on Cancer and Aging, Nice (IRCAN), Cote d’Azur University, 06000 Nice, France; LASSALLE.S@chu-nice.fr (S.L.); hofman.p@chu-nice.fr (P.H.); 3 Biobank BB-0033-00025, FHU OncoAge, IRCAN, Laboratory of Clinical and Experimental Pathology, University Hospital of Nice, 06000 Nice, France; 4 Department of Dermatology, Archet 2 Hospital, 151 Route de Saint-Antoine, 06200 Nice, France; picard-gauci.a@chu-nice.fr (A.P.-G.); montaudie.h@chu-nice.fr (H.M.); 5 Department of Oncology, Antoine Lacassagne Cancer Centre, 06000 Nice, France; lauris.GASTAUD@nice.unicancer.fr; 6 Department of Biology and Pathologies of Melanocytes, Team1, Equipe Labellisée Ligue 2020 and Equipe Labellisée ARC 2019, Centre Méditerranéen de Médecine Moléculaire, Inserm, 06200 Nice, France; Corine.Bertolotto@unice.fr; 7 Cervicofacial Surgery Department, Antoine Lacassagne Cancer Centre, 06000 Nice, France; gilles.poissonnet@nice.unicancer.fr

**Keywords:** periocular malignant tumours, orbital exenteration, targeted therapy, immunotherapy, eye-sparing

## Abstract

**Simple Summary:**

Managing locally advanced periocular malignant tumours, especially those invading the orbit, is challenging. Orbital exenteration has long been considered the gold standard. The development of conservative surgical techniques in the early 2000s, followed by the emergence of new targeted therapies and immunotherapies over the past decade, has led to a paradigm shift towards the use of ‘eye-sparing’ strategies.

**Abstract:**

The management of periocular skin malignant tumours is challenging. Surgery remains the mainstay of treatment for localised eyelid cancers. For more locally advanced cancers, especially those invading the orbit, orbital exenteration has long been considered the gold standard; however, it is a highly disfiguring and traumatic surgery. The last two decades have been marked by the emergence of a new paradigm shift towards the use of ‘eye-sparing’ strategies. In the early 2000s, the first step consisted of performing wide conservative eyelid and orbital excisions. Multiple flaps and grafts were needed, as well as adjuvant radiotherapy in selected cases. Although being incredibly attractive, several limitations such as the inability to treat the more posteriorly located orbital lesions, as well as unbearable diplopia, eye pain and even secondary eye loss were identified. Therefore, surgeons should distinguish ‘eye-sparing’ from ‘sight-sparing’ strategies. The second step emerged over the last decade and was based on the development of targeted therapies and immunotherapies. Their advantages include their potential ability to treat almost all tumours, regardless of their locations, without performing complex surgeries. However, several limitations have been reported, including their side effects, the appearance of primary or secondary resistances, their price and the lack of consensus on treatment regimen and exact duration. The aim of this article was to review the evolution of the management of locally advanced periocular malignant tumours over the last three decades and highlight the new paradigm shift towards the use of ‘eye-sparing’ strategies.

## 1. Introduction

**Figure 1:**
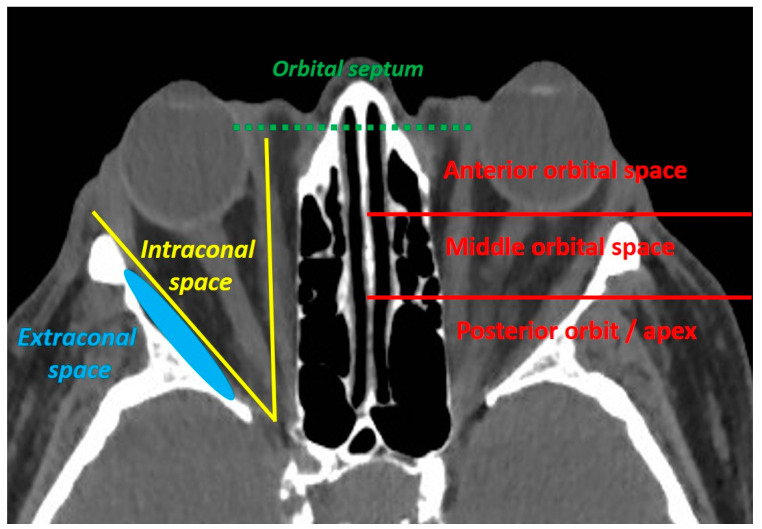
The orbital invasion (defined as an orbital septum violation) by an eyelid malignant tumour can be defined as intraconal (if located inside the oculomotor muscle cone) or extraconal (if located outside the oculomotor muscle cone), and should be located according to its depth (anterior, middle or posterior orbit).

The eyelids are considered a high-risk skin malignancy area. Managing periocular tumours is challenging for functional and cosmetic reasons. Basal cell carcinoma (BCC) is the most common eyelid cancer, followed by squamous cell carcinoma (SCC), melanoma, sebaceous carcinoma and Merkel cell carcinoma (MCC) [1]. Surgery remains the mainstay of treatment for localised tumours, with the aim of obtaining clear surgical margins. Tumours originating from the internal or external canthus are at particular risk of orbital invasion [2,3]. An orbital involvement is defined as an orbital septum violation by the tumour. The orbital invasion should be classified as anterior, middle or posterior, and the extraconal or intraconal involvement should be specified (Figure 1).

Until recently, an eyelid malignancy invading the orbit was considered an indication for orbital exenteration (OE). However, OE is a radical, disfiguring and psychologically traumatic surgical procedure often refused by patients [4]. In addition, OE cannot be offered to one-eyed patients. Therefore, several authors have tried to develop ‘eye-sparing’ strategies based on conservative surgical techniques followed or not by radiotherapy [2]. Although being attractive, conservative combined eyelid and orbital surgeries have been associated with several post-operative complications, limiting their interest [4]. In addition, several patients have experienced vision loss, and secondary eye amputation was sometimes required [2,3]. Therefore, a distinction between ‘eye-sparing’ and ‘sight-sparing’ strategies has emerged [4]. Over the last decade, targeted therapies such as anti-SMO (smoothened protein) therapies for the treatment of BCC have emerged as a viable strategy for locally advanced periocular malignant tumours. These new targeted therapies and immunotherapies have opened a new era towards personalised periocular cancer treatment.

The aim of this review was to summarise the evolution of the management of periocular malignant tumours over the last three decades and highlight the current paradigm shift towards the use of ‘eye-sparing’ strategies.

## 2. Method for Literature Search

A thorough literature search was performed on Medline (https://pubmed.ncbi.nlm.nih.gov/) over the 2001–2021 period using the main search term ‘(orbital exenteration) or (periocular tumors)’ and the following terms: ‘eye sparing’, ‘globe sparing’, ‘targeted therapy’ and ‘immunotherapy’. Title and abstracts were reviewed by two independent authors. References were also obtained from citations in papers identified in the original search. Only relevant articles focused on eye-sparing strategies (e.g., conservative surgery, orbital radiotherapy, targeted therapy or immunotherapy) and written in English or French were considered. A few select articles published before 2001 were included in the text for historical and didactic purposes; however, the review was mainly based on articles published over the past 2 decades.

## 3. Orbital Exenteration for Locally Advanced Periocular Malignant Tumours

**Figure 2:**
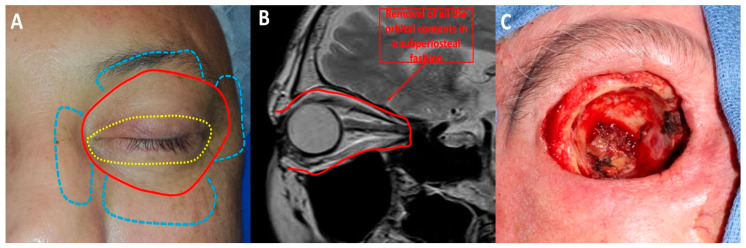
Orbital exenteration: (**A**) Several techniques have been described: eyelid-sparing orbital exenteration (yellow), total orbital exenteration (red) and orbital exenteration extended to surrounding orbital structures (blue). (**B**) Orbital exenteration consists of removing all the orbital contents. (**C**) Intraoperative photograph of a case of total orbital exenteration.

**Figure 3:**
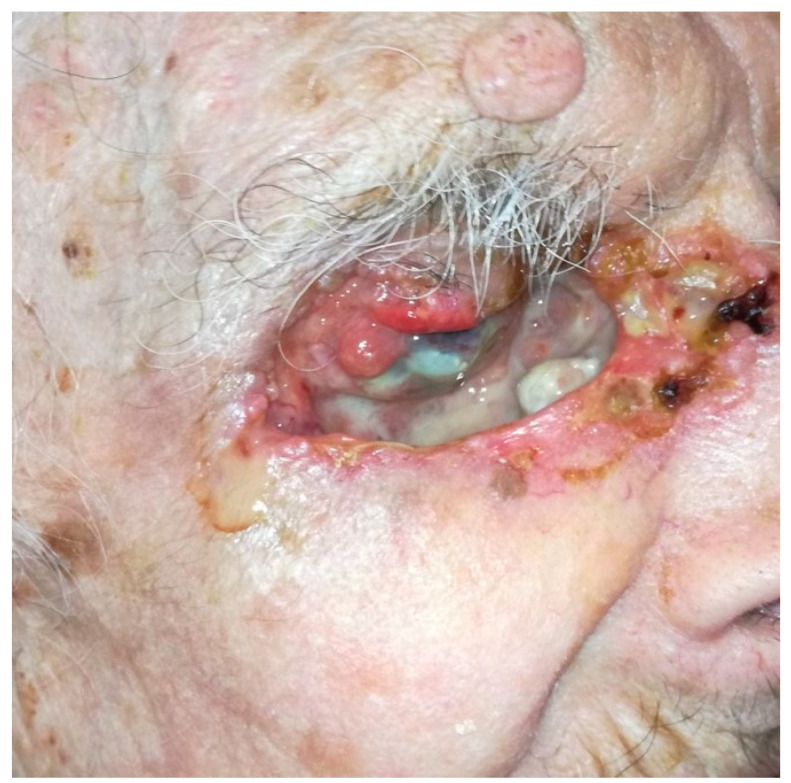
‘Pseudo-orbital exenteration’ of an eyelid BCC with orbital invasion.

**Figure 4:**
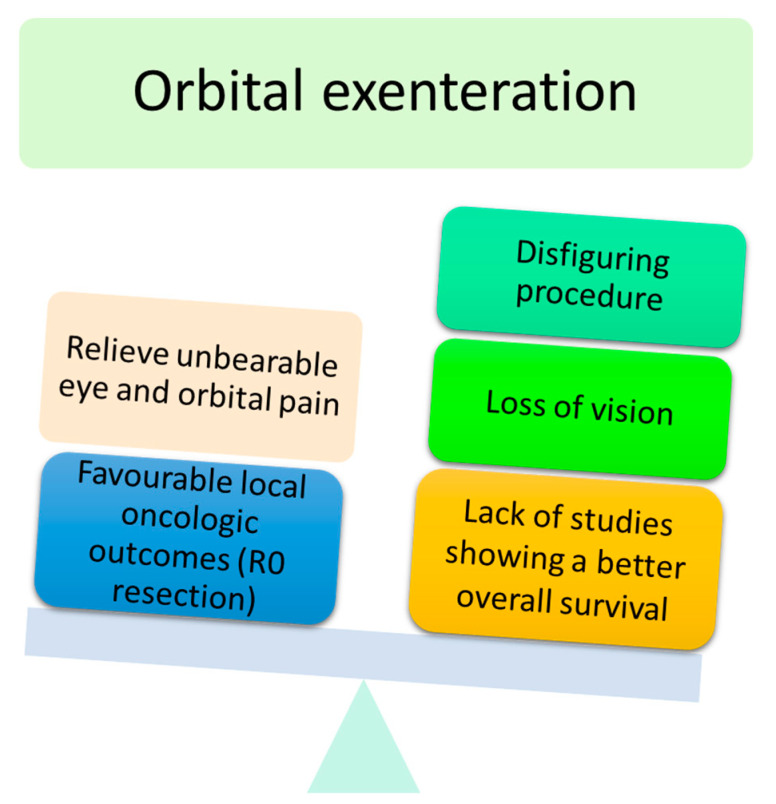
Main advantages and disadvantages of orbital exenteration.

OE is a radical surgical procedure consisting of the removal of the entire orbital contents, including the eye and oculomotor muscles, in a subperiosteal fashion (Figure 2) [5]. Historically, OE was first described in 1583 by Bartisch et al. [6]. Depending on the tumour location and extent, OE may be enlarged to the adjacent sinus cavities or anterior cranial fossa. Reconstruction differs depending on the surgeon’s speciality and ranges from spontaneous granulation of the orbital socket to more complex and time-consuming free flaps [7]. Cosmetic rehabilitation is better achieved with an orbital prosthesis retained by orbital implants, skin glue or glasses [4]. Cosmetic rehabilitation depends on orbital socket healing and is often delayed, especially in the case of orbital implant placement [5]. Although recent progress has been made in terms of reconstructive strategies and cosmetic rehabilitation [4], OE is associated with anxiety and depression [8]. Periocular eyelid malignant tumours invading the orbit are the most common indication for OE [9]. BCC is one of the most common eyelid malignant tumours invading the orbit. Although BCC virtually does not metastasise, it is associated with local aggressiveness, as shown in Figure 3. Other potential metastatic malignant tumours, such as SCC, melanoma or lacrimal gland tumours, often require OE. To date, no studies with a high level of evidence have shown the benefit of OE compared with conservative surgery in terms of overall survival [4]. The advantages and disadvantages of OE are shown in Figure 4.

Ophthalmologists have to deal with a very psychologically and anatomically traumatic surgery, which is sometimes refused by patients and cannot be performed in one-eyed patients. Therefore, several authors have tried to develop more conservative strategies called ‘eye-sparing’ strategies (Figure 2).

## 4. First Step towards Eye-Sparing Strategies: Conservative Surgery Followed or Not by Adjuvant Radiotherapy

In 2005, Leibovitch et al. [2] were the first to introduce the concept of ‘eye-sparing’ strategies by reporting their experience with 64 BCC patients with orbital invasion. Of these 64 patients, 16 were not treated with OE due to patient’s refusal, one-eyed patients or unresectable tumours (intraconal or posterior orbital location). These 16 patients were treated with conservative surgery alone, radiotherapy alone or a combination of both [2]. Tumour recurrence was found in 2.8%, 16.7% and 25% of patients treated with OE, surgical excision alone and radiotherapy alone, respectively. They found that about 25% of patients treated with radiotherapy developed mild side effects such as dry eye syndrome or mild radiation retinopathy. They concluded that, in highly selected patients (e.g., one-eyed patients and patients with anterior and extraconal orbital involvement), an eye-sparing strategy could be an alternative to OE.

In 2010, Madge et al. [3] have published the results of a multicentric international study assessing the outcomes of conservative eye-sparing surgery in 20 patients with locally advanced eyelid BCC. Only patients with anterior orbital invasion were included. All the tumours originated from the medial canthal area. Conservative surgery consisted of wide tumour and lacrimal sac resection guided by rapid paraffin or frozen section histological margin control followed by local and/or regional flaps. Complete surgical excision (R0 resection) was achieved in 90% of patients. Adjuvant orbital radiotherapy was performed in the two (10%) patients with positive surgical margins. After a mean clinical and radiological follow-up of 2 years, only one (5%) patient experienced tumour recurrence and, thus, underwent OE. Despite these favourable oncological outcomes, enthusiasm must be tempered. Indeed, 60% of patients experienced post-operative restrictive diplopia related to reduced medial rectus motility. Among them, three patients experienced diplopia in the primary gaze and one wore an eye patch to relieve double vision. Permanent epiphora was diagnosed in 75% of patients. About 60% of patients underwent a subsequent surgical revision for conjunctival, eyelid or lacrimal disorders. About 85% of patients had a stable visual acuity throughout the study. For the first time, this study reported excellent oncological outcomes and visual preservation after conservative surgery. However, the post-operative complications, high rate of surgical revisions and need for a close clinical and radiological follow-up should be taken into account, especially in elderly patients.

**Figure 5:**
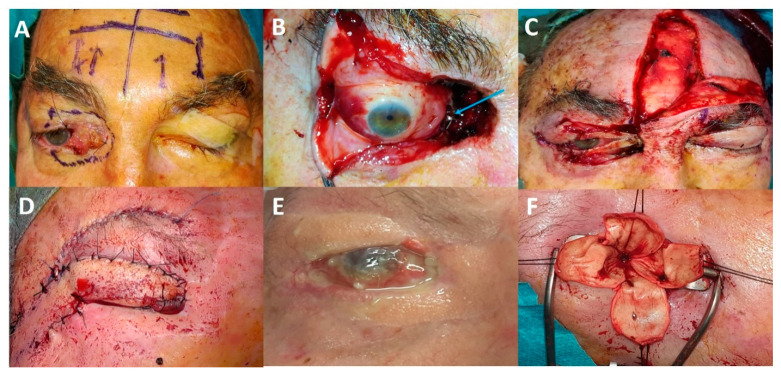
Illustrative case of eye-sparing surgery: (**A**) A 68-year-old patient with upper and lower eyelid squamous cell carcinoma with anterior and extraconal orbital involvement. (**B**) Removal of half of the upper and lower eyelids, lacrimal sac and tantalum clip placement for adjuvant proton beam therapy (blue arrow). (**C**) Reconstruction performed using a tarsal graft, a conchal graft and a frontalis muscle flap. (**D**) Second surgery: frontotemporal flap (Fricke flap) used to correct the upper eyelid retraction and subsequent corneal exposure. (**E**) Flap retraction associated with chronic painful corneal ulcer. (**F**) Third surgery: eye evisceration to relieve unbearable eye pain.

Data on eye-sparing surgery in more aggressive eyelid malignant tumours, such as SCCs or sebaceous carcinomas, are limited. In our experience with eyelid SCC invading the anterior orbit, achieving clear surgical margins is more challenging due to the invasive nature of the tumour (Figure 5). Adjuvant radiotherapy is more likely to increase the rate of complications such as eyelid retraction, lagophthalmos, severe keratitis, dry eye, neovascular glaucoma and optic neuropathy [4,10]. Several authors have used eye-sparing strategies (conservative surgery plus adjuvant photon or particle radiotherapy) for the treatment of lacrimal gland or sinus carcinomas [11,12,13]. Between 10% and 50% of patients experienced a visual decrease over time. In certain circumstances, patients may experience a complete visual loss and unbearable eye pain. Such patients often ask for eye amputation to improve their quality of life (Figure 5). Finally, adjuvant orbital radiotherapy is known to impair orbital socket healing in the case of secondary OE.

**Figure 6:**
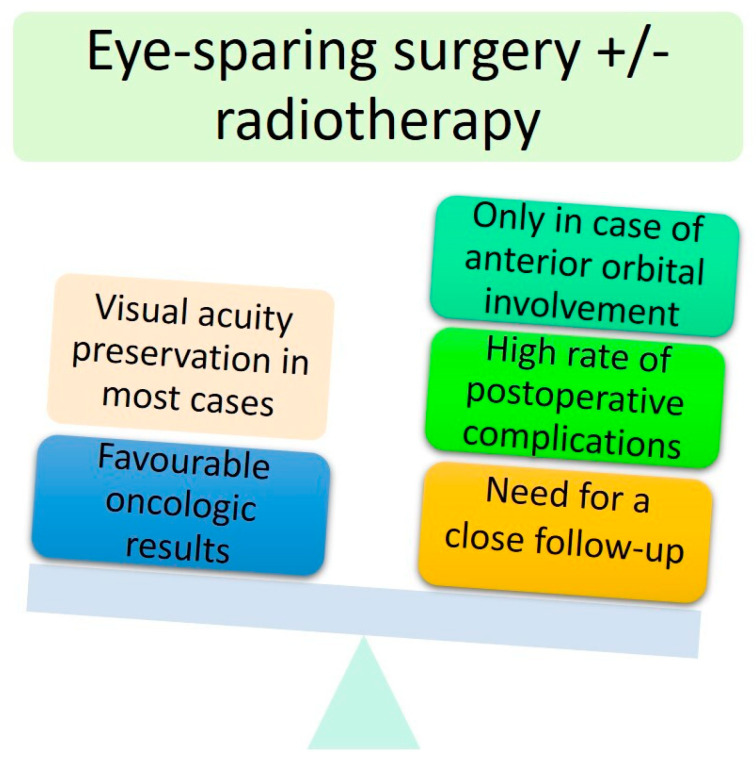
Main advantages and disadvantages of conservative surgery.

To conclude, eye-sparing strategies appear to be a viable procedure for locally advanced periocular malignant tumours with anterior and extraconal orbital involvement, especially in one-eyed patients. However, most patients will experience post-operative complications, and subsequent surgical revision will be needed with the risk of a significantly reduced quality of life. OE remains the mainstay of treatment for more posteriorly located tumours (intraconal middle and posterior tumours). For more aggressive malignant tumours (SCC and sebaceous carcinoma), the need for adjuvant orbital radiotherapy will probably worsen the visual impairment. Therefore, it is essential to distinguish ‘eye-sparing’ from ‘sight-sparing’ strategies [4]. The advantages and disadvantages of ‘eye-sparing’ strategies are summarised in Figure 6.

## 5. Second Step towards Eye-Sparing Strategies: Use of Targeted Therapies and Immunotherapies

### 5.1. Targeted Therapies in Locally Advanced BCC: More Questions Than Answers?

The first revolution occurred in 2012 when anti-SMO targeted therapies emerged as a viable treatment for locally advanced BCC [14]. About 90% of BCCs carry a disactivating mutation in the *PTCH1* gene. This mutation results in an overactivation of the Hedgehog signalling pathway via the SMO receptor, leading to an anarchic cell proliferation that ultimately results in BCC. Vismodegib and sonidegib are two anti-SMO therapies approved by the FDA. Recently, anti-SMO therapies have been used for the treatment of ‘locally advanced’ periocular BCC. These studies are briefly summarised in Table 1. This table allows for a better understanding of the current limitations and lack of clear guidelines for anti-SMO therapies in periocular BCC.

It is interesting to note the following:

- Between 58% and 75% of patients had an orbital involvement at the time of diagnosis. For these patients, anti-SMO therapies were prescribed to avoid OE. However, for the remaining patients without obvious orbital involvement, anti-SMO therapies were used to treat large eyelid tumours for which a wide surgical excision followed by multiple grafts and flaps would have otherwise been required. In addition, extended periocular reconstruction is often associated with residual eyelid malposition with subsequent exposure to keratitis [20]. Taken together, these data indicate that the term ‘locally advanced’ can be used for either eyelid tumours invading the orbit or eyelid tumours requiring complex and disfiguring periocular flaps and grafts.
- Treatment duration was not consensual and ranged from 2 to 53 months. This highlights the lack of consensus on treatment regimen (daily versus sequential administration) and exact duration. Sequential treatment with drug discontinuation during the weekend is currently under investigation.
- The rate of complete response was highly variable, ranging from 29% to 67%. This could be explained by the lack of clear guidelines for the definition of remission. Several studies were based on a clinical examination (mainly based on the RECIST criteria [15,17]), whereas other studies were based on a systematic surgical biopsy [16]. Of course, the histological analysis remains the gold standard, and the benefit/risk ratio and economic balance should be carefully considered for each patient.
- The large differences in the rates of patients undergoing adjuvant surgical excision (ranging from 4.7% to 100%) highlight two radically opposed treatment paradigms. Should anti-SMO therapies be considered a neoadjuvant treatment allowing surgeons to reduce perioperative surgical morbidity or a cure (i.e., treatment to be carried out until complete tumour removal)? The neoadjuvant paradigm would have several advantages such as reducing treatment duration (through reduced cost and treatment side effects) and systematic histopathological whole tumour control. To our knowledge, no study has investigated the use of anti-SMO therapies as an adjuvant treatment in the case of R1 or R2 periocular BCC resection.
- Up to 13% of patients experienced tumour progression despite treatment administration. This could be related to an acquired primary or secondary resistance to treatment [21], as seen observed in other BCC locations [22].
- Anti-SMO side effects (i.e., alopecia, nausea, dysgeusia and muscle spasms) are known to be highly prevalent, affecting about 100% of patients. While they were often considered mild, drug discontinuation was needed in 7.7–38% of patients in periocular BCC studies. For these patients, systematically performing a biopsy after treatment should be discussed to ensure complete tumour removal. This high treatment discontinuation rate highlights the need to develop an alternative treatment regimen (discontinuation regimen). In addition, serious anti-SMO side effects should not be neglected. In their study involving 244 periocular BCCs, [19] found that 5.7% of patients died from anti–SMO-related side effects, whereas only 2% died from disease progression [19]. This finding should be kept in mind, especially in more fragile and elderly patients.
- Finally, the most meaningful data are probably the rate of secondary OE, ranging from 0% to 23%. As previously stated, the goal of anti-SMO therapies is eye preservation by avoiding OE. Unsurprisingly, this rate was higher in studies with a longer follow-up [15,18]. This result shows that anti-SMO therapies are not the ‘holy grail’.

Although they have shown interesting results in the management of periocular BCC, preliminary studies assessing anti-SMO therapies raise more questions than they provide answers. Clear guidelines are currently lacking, and further information on treatment duration and regimen is needed. More importantly, the underlying treatment paradigm should be clarified (i.e., neoadjuvant versus curative treatment). Several studies have found that the most important side effects of anti-SMO therapies usually appeared after about six months of treatment [9,15]. Prescribing targeted therapies as a neoadjuvant treatment for 6 months, followed by systematic surgery, could be an interesting treatment protocol that could be proposed.

**Figure 7:**
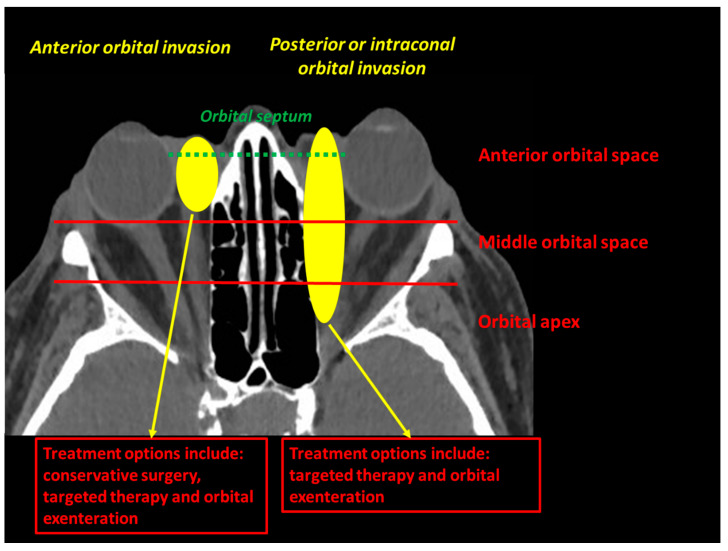
Current treatment options for locally advanced BCC invading the anterior orbit versus the middle/posterior orbit. Targeted therapies might be used to treat both posteriorly located and intraconal orbital BCC.

Undoubtedly, a major advantage of targeted therapies is their theoretical ability to treat the untreatable conservatively. For example, a BCC invading the middle or posterior orbit or the intraconal orbital space would never be accessible to conservative surgery (Figure 7). Before the anti-SMO therapy era, only OE would have been proposed to such patients. Further studies are needed to confirm this theory.

Finally, anti-SMO therapies are especially useful for the treatment of multifocal BCC, as found in Gorlin syndrome.

### 5.2. Targeted Therapies Used for Other Periocular Malignant Tumours

Despite recent knowledge and new insights based on biological and genetic findings, data on periocular malignant tumours and targeted therapies are still limited (except for BCC). The main molecular targets are presented according to tumour histology in Table 2.

Eyelid SCC accounts for about 5–10% of eyelid malignant tumours [23] and may originate from the skin or conjunctiva. Several studies have shown that in both cutaneous and conjunctival SCC, the EGFR (epithelial growth factor receptor) was overexpressed [24]. In most cases, a wide surgical excision followed or not by adjuvant radiation beam therapy is advocated. Several case series have reported favourable outcomes when EGFR inhibitors were used for the treatment of locally advanced eyelid SCC in order to avoid OE [25,26] and the treatment of metastatic SCC. Further studies are currently ongoing.

The last decade has been marked by a dramatic improvement in cutaneous melanoma prognosis. Anti-BRAF and anti-MEK targeted therapies prescribed in combination have revolutionised the treatment of BRAF-mutated cutaneous melanoma [27]. Recently, several studies have shown that conjunctival melanoma shared molecular similarities with cutaneous melanoma [28,29]. Unlike uveal melanoma, about half of the conjunctival melanomas have been shown to carry *BRAF*, *KRAS*, *NRAF* and *NF1* mutations [30,31]. At the time of writing this article, only a few case series have reported favourable oncological outcomes with anti-BRAF alone, anti-MEK alone or a combination of both in locally advanced and metastatic conjunctival melanoma [32,33]. Currently, determining the *BRAF* mutational status is a standard of care in eyelid (cutaneous or conjunctival) melanoma [34].

Eyelid sebaceous carcinoma is a rare periocular malignant tumour. A wide local surgical excision (surgical margins >1 cm) with intraoperative histological margin control is recommended [35]. This implies total or subtotal eyelid removal. To date, no clinical study has reported the use of targeted therapies in eyelid sebaceous carcinoma. A recent study has found that the Hedgehog pathway was upregulated in sebaceous carcinoma [36]. This could support the use of anti-SMO therapies as in BCC. Other studies have found potentially targetable dysregulations in the HER2 and Pi3K signalling pathways [37,38].

MCC is a rare but extremely aggressive malignant tumour. A wide surgical excision is the mainstay of treatment, sometimes associated with sentinel lymph node biopsy [39]. However, despite adequate management, many patients will develop metastases. In 2008, the presence of Merkel cell polyomavirus in MCC was discovered, leading to the distinction between virus-positive and virus-negative MCC [39]. This distinction is relevant, especially when immunotherapy is considered (see below). Several targetable pathways have also been identified, such as the AKT-mTOR pathway [40]. To date, there is no standard of care, and treatment mainly depends on tumour sequencing analyses.

### 5.3. Immunotherapies

Immunotherapy has gained incredible popularity in the treatment of periocular malignant tumours. The underlying mechanism is to allow the immune system to attack hidden cancer cells. The most common immune checkpoint inhibitors are anti–PD-1 (programmed cell death-1) and anti-CTLA4 (cytotoxic T-lymphocyte antigen-4) therapies that may be prescribed alone or in combination. Immunotherapy is more likely prescribed in the case of high tumour mutational burden.

Among cutaneous malignant tumours, melanoma has been the first tumour to show a clinical benefit due to immunotherapy progress. Several studies have shown that PD-L1 was expressed in cutaneous and conjunctival melanomas [41,42]. A pivotal study published in 2010 found, for the first time, an improved survival in patients with metastatic cutaneous melanoma treated with ipilimumab [43]. Recent studies have reported encouraging results when immunotherapy was prescribed for locally advanced and metastatic eyelid cutaneous and conjunctival melanomas [43]. Moreover, in two case series involving 10 patients with conjunctival melanoma, half of the patients achieved a complete response and half had a stable disease [44,45]. In some instances, immunotherapy has allowed the need for OE to be avoided [4], and could be considered a neoadjuvant treatment [4].

Eyelid SCC is also a good candidate for immunotherapy given its high intrinsic mutational burden. Several studies have reported an overexpression of PD-L1 in cutaneous and conjunctival SCC [41]. Immunotherapy has been shown to be effective in cutaneous and head and neck metastatic SCC [41,46]. A recent article reported the case of a patient with locally advanced periocular SCC invading the orbit and skull base who was successfully treated with a PD-1 inhibitor [47]. These findings would allow immunotherapy to be considered an eye-sparing strategy in highly selected cases.

Immunotherapy has also been investigated in MCC. Immunogenic MCC (i.e., virus-positive MCC or virus-negative MCC with high tumour mutational burden) is more likely to be accessible to immunotherapy [40]. Encouraging results have been reported with anti-PD-L1 therapies [48], and other clinical trials are currently ongoing. Interestingly, several authors have investigated the abscopal effect of the combination of local radiotherapy and immunotherapy [40].

Similarly, PD-L1 has been found to be overexpressed in about half of eyelid sebaceous carcinomas [49,50]. To date, only isolated case reports have suggested the efficacy of anti-PD-1 immunotherapy in metastatic sebaceous carcinomas [51].

**Figure 8:**
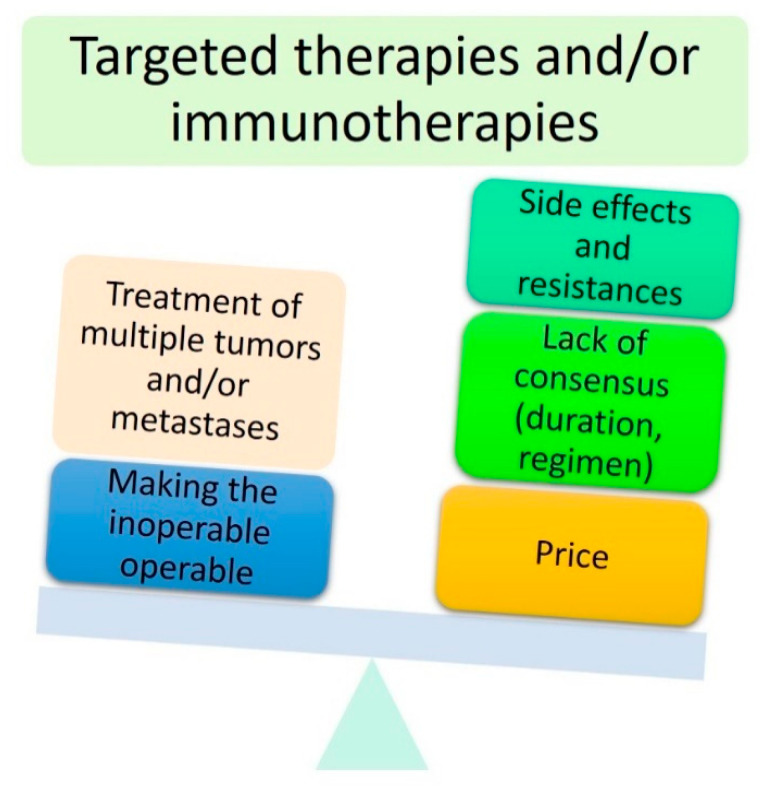
Main advantages and disadvantages of targeted therapies and immunotherapies.

Except for BCC, the prescription of targeted therapies or immunotherapies for locally advanced periocular malignant tumours remains occasional. Most studies have investigated these new molecular treatments in the context of a metastatic disease. Through the accumulation of data over time, it may be assumed that these new treatments might be considered neoadjuvant treatments, allowing for eye preservation or leading to reduced surgical morbidity. They will undoubtedly allow physicians to offer a personalised treatment to each patient. The advantages and disadvantages of targeted therapies and immunotherapies are summarised in Figure 8.

## 6. Conclusions

Does OE still have a place in the management of periocular malignant tumours in this new era of eye-sparing strategies? According to us, OE remains a standard of care for eyelid malignant tumours invading the middle orbit, posterior orbit, intraconal space and in the case of unbearable orbital pain. Although being extremely attractive, conservative surgical approaches are not suitable for posteriorly located orbital cancers and may be associated with several complications that may compromise vision. Therefore, a distinction should be made between the terms ‘eye-sparing’ and ‘sight-sparing’. Recent targeted therapies and immunotherapies have the advantage of treating almost all lesions, regardless of their location. However, not all patients are eligible for such therapies and the lack of consensus, appearance of a secondary resistance and price considerations deserve further investigation.

## Figures and Tables

**Table 1:** Main studies that assessed anti-SMO targeted therapies in locally advanced periocular BCC.

Author, Year	Number of Patients	Number (%) of Patients with Orbital Involvement	Mean (Range) Treatment Duration (Months)	Number (%) of Patients Achieving a Complete Response	Number (%) of Patients Achieving an Incomplete Response	Number (%) of Patients with a Progressive Disease	Number (%) of Patients Undergoing Adjuvant Surgical Excision	Number (%) of Patients Undergoing Secondary Orbital Exenteration	Number (%) of Patients Who Discontinued Treatment Due to Excessive Side Effects (%)	Mean (Range) Follow-Up (Months)
Wong, 2015 [15]	15	10 (67)	13 (2–40)	10 (67)	3 (20)	2 (13)	1 (7)	3 (20)	5 (33)	36 (14–52)
Sagiv, 2018 [16]	8	6 (75)	14 * (4–36)	5 (62.5) ^†^	3 (37.5)	0 (0)	8 (100)	0 (0)	2 (25)	18 (6–43)
Eiger-Moscovich, 2019 [17]	21	15 (71.5)	9 * (1–53)	10 (48)	11 (52)	0 (0)	1 (4.7)	1 (4.7)	8 (38)	26 * (9–60)
Oliphant, 2020 [18]	13	7 (58)	7 (2–36)	5 (38)	8 (54)	0 (0)	6 (46)	3 (23)	1 (7.7)	30 (12–48)
Ben Ishai, 2020 [19]	244 ^‡^	NR	10 * (5–19.5)	70 (28.7)	94 (38.5)	5 (2)	NR	NR	58 (23.8)	10 * (5.7–14)

* median; † histologically proven, ‡ post hoc analysis of the STEVIE trial; NR: not reported.

**Table 2:** Main targeted therapies and immunotherapies according to tumour type.

Tumour Histology	Main Molecular Target
BCC	Hedgehog pathway (SMO)
SCC	EGFR PD-1/PD-L1
Melanoma (lid or conjunctiva)	BRAF PD-1/PD-L1 CTLA4
Sebaceous carcinoma	Hedgehog pathway HER2 Pi3K pathway PD-1/PD-L1
Merkel cell carcinoma	AKT-mTOR pathway PD-1/PD-L1
